# μ-Slide Chemotaxis: A new chamber for long-term chemotaxis studies

**DOI:** 10.1186/1471-2121-12-21

**Published:** 2011-05-18

**Authors:** Pamela Zengel, Anna Nguyen-Hoang, Christoph Schildhammer, Roman Zantl, Valentin Kahl, Elias Horn

**Affiliations:** 1 Department of Otorhinolaryngology, Head and Neck Surgery, Grosshadern Medical Centre, Ludwig-Maximilians-University of Munich, Marchioninistr. 15, 81377 Munich, Germany; 2 Hochschule Weihenstephan-Triesdorf, 85350 Freising, Germany; 3 ibidi GmbH, Am Klopferspitz 19 D-82152 Martinsried Munich, Germany

## Abstract

**Background:**

Effective tools for measurement of chemotaxis are desirable since cell migration towards given stimuli plays a crucial role in tumour metastasis, angiogenesis, inflammation, and wound healing. As for now, the Boyden chamber assay is the longstanding "gold-standard" for in vitro chemotaxis measurements. However, support for live cell microscopy is weak, concentration gradients are rather steep and poorly defined, and chemotaxis cannot be distinguished from migration in a single experiment.

**Results:**

Here, we describe a novel all-in-one chamber system for long-term analysis of chemotaxis in vitro that improves upon many of the shortcomings of the Boyden chamber assay. This chemotaxis chamber was developed to provide high quality microscopy, linear concentration gradients, support for long-term assays, and observation of slowly migrating cells via video microscopy. AlexaFluor 488 dye was used to demonstrate the establishment, shape and time development of linear chemical gradients. Human fibrosarcoma cell line HT1080 and freshly isolated human umbilical vein endothelial cells (HUVEC) were used to assess chemotaxis towards 10% fetal calf serum (FCS) and FaDu cells' supernatant. Time-lapse video microscopy was conducted for 48 hours, and cell tracking and analysis was performed using ImageJ plugins. The results disclosed a linear steady-state gradient that was reached after approximately 8 hours and remained stable for at least 48 hours. Both cell types were chemotactically active and cell movement as well as cell-to-cell interaction was assessable.

**Conclusions:**

Compared to the Boyden chamber assay, this innovative system allows for the generation of a stable gradient for a much longer time period as well as for the tracking of cell locomotion along this gradient and over long distances. Finally, random migration can be distinguished from primed and directed migration along chemotactic gradients in the same experiment, a feature, which can be qualified via cell morphology imaging.

## Background

Chemotaxis has been a focus of research for more than a century due to its involvement in several important physiological and pathological processes such as tumour metastasis [1,2], angiogenesis [3], inflammation [4], arteriosclerosis [5], and many other processes of great interest to biomedical research. For example, neo-angiogenesis is controlled by the production of chemotactic factors, which trigger the migration of endothelial cells into the tumour tissue. The formation of new blood vessels is mandatory for the proliferation of cancer, since tumours greater than 1.5 mm^3 ^in size require intimate contact to blood vessels for nutrition supply to avoid necrosis [6]. Chemotaxis is obligatory beyond neo-angiogenesis in initial steps of malignant transformation. During the process of tumour cell dissemination, transformed cells depend upon migration in order to seed themselves in novel tissue and thereby form metastasis. Likewise, during the inflammatory response, immune cells migrate from the periphery to an injury site in response to locally released chemotactic agents [7]. Although this process is beneficial under normal circumstances, negative consequences can occur if this inflammatory response becomes chronic [8,9]. In order to identify pharmaceuticals that can effectively modulate this immune response, it would be beneficial to have a sensitive and reproducible assay to test potential drugs *in vitro *[10,11].

Although there are several methods to measure chemotaxis *in vitro*, very different methods have become pervasive. The first system is the Boyden chamber [12-14] and derived assays that work with either thick filters or thin porous membranes. In these assays, the cells are placed on a microporous membrane above a chemotactic agent. In response to a concentration gradient of chemotactic agent, cells migrate through the membrane to the lower reservoir. Migrating cells can then be counted on the reverse side of the membrane after staining, usually as an endpoint assay at a predetermined time. These chemotaxis assays are widespread; however, the information obtained is limited, as live cell microscopy is substantially restricted, and gradients are very steep and rather undefined. Although this technique allows for the performance of many simultaneous assays in parallel, it also has many limitations and drawbacks: In particular, the counting of migrated cells can be time consuming, tedious, and subject to error. Furthermore, the very steep and transient nature of the gradient only models the conditions experienced by cells *in vivo *that might appear at vessel walls. The Boyden assays do not access the cells'paths or locomotion, and persistent chemotaxis cannot be distinguished from random migration in one single experiment; thus, separate controls are required.

The second important assay is based on the Zigmond chamber [15] and its derivates [16], which do provide defined linear gradients that reach steady-state levels along with better microscopy properties. Although suitable for time-lapse microscopy, Zigmond chambers are suboptimal with respect to long-term stability and convenience of handling, which render the observation of slowly migrating cells virtually impossible. The chambers have to be cleaned, sterilized and assembled, which is undesirable for maximum reproducibility. Finally, modern time-lapse microscopy generally operates with inverted microscopes for which the chamber is not designed.

Several additional techniques for measuring chemotaxis in vitro are possible as microfluidic devices, which offer further capabilities for examining chemotaxis in detail. However, the usage of these techniques is very complex [17-19]. The advantages of microfluidic devices for chemotaxis studies include a fast and reliable setup of the gradients and the possibility to alter the direction of either the gradient or the steepness over time. Regarding steady flow conditions and the related consumption of chemoattractant solutions, this device is more suited for fast-migrating cells like neutrophils [20]. The associated requirement for flow-producing equipment also makes handling complicated. In order to keep the gradient stable over time, continuous flow is needed, which may result in an overlay of effects between chemotaxis and flow-mediated activation of the cell cytoskeleton in reaction against the shear stress that may be inadvertently applied.

Here, we describe a new chamber that improves on many of these shortcomings; it allows for long-term analysis of chemotaxis as well as the observation of slow migrating mammalian cells. The one-piece system is also disposable, which eliminates all problems associated with cleaning. Furthermore, it is designed for inverted microscopes, thereby integrating chemotaxis and high resolution microscopy in a convenient way for the very first time. Using the AlexaFluor 488 dye, we demonstrated a linear and steady gradient within the chamber, which resulted in excellent and reproducible results with respect to measurement of chemotaxis in various settings. Hence, the presented novel chamber is a valuable tool for both research and drug development.

## Methods

### Fluorescence microscopy and live cell imaging

Fluorescence measurements were performed using an inverted confocal Axiovert 200/LSM 510 microscope equipped with a 63 ×/1.4 oil plan-apochromat objective (Carl Zeiss, Jena, Germany). In order to reduce out-of-focus blur resulting from the different heights, the confocal pinhole was set to one airy unit leading to an optical slice of <0.7 μm. Measurements were taken 20 μm above the bottom. Single scan fields were combined to scan a large area of 2.194 μm × 293 μm. AlexaFluor 488 dye (Invitrogen, Carlsbad, USA) was used to measure concentration profiles in water at 22°C. For fluorescence excitation, the argon laser line 488 nm and a standard filter set were used. AlexaFluor 488 dye (diffusion coefficient D = 430 μm^2^/s [21] was used for both chemoattractant-free and chemoattractant-containing medium as described below. Concentrations of 10 μM, which represented an empirical minimum concentration (=C_0_), and 100 μM were used. These two concentrations were chosen in order to visualize the dynamic linear range of the fluorescence to be measured.

Time-lapse video microscopy with cells was performed using an inverted microscope Eclipse Ti (Nikon, Düsseldorf, Germany), a 4x phase contrast objective, and a CCD camera (DS-Qi1MC, Nikon, Düsseldorf, Germany) with a time interval of ten minutes between images. The slide was inserted into a 37°C heating and incubation system (ibidi GmbH, Martinsried, Germany) consisting of a heated ground plate in 96 well plate and inserts adapted to the chamber's geometry. A heated lid made from ITO glass prevented condensation effects within the incubation system. The system was flushed with actively mixed 5% CO_2 _at a rate of ten l/hour.

### Cell culture

Human fibrosarcoma epithelial cell line HT-1080 (obtained from Toni Lindl, Munich, Germany, ATCC No.: CCL-121) was grown in Dulbecco's modified Eagle's Medium (DMEM; PromoCell, Heidelberg, Germany) supplemented with 10% fetal calf serum (FCS; Sigma-Aldrich) at 37°C and 5% CO2. HUVEC 1 (Human Umbilical Vein Endothelial Cell) were freshly isolated from human umbilical veins of newborn babies by collagenase digestion, as previously described [22,23]. All patients with no special risks before giving birth were routinely asked for their consent for using anonymous tissue materials for non-profit research. Since no risk or additional encumbrance was induced by collecting the umbilical cords, the local ethics committee did not need to be informed in this case.

HUVEC 2 were bought from Lonza, Verviers, Belgium. HUVEC were grown in monolayers, harvested by centrifugation, and amplified at 37°C in endothelial cell growth medium 2 (EBM2, Cambrex, Verviers, Belgium). In all experiments, only the first three passages of HUVEC 1 and passages three to five of HUVEC 2 were used. Cells were grown to 80-90% confluence, trypsinised, and filled into the chemotaxis chambers at a density of 3 × 10^6 ^cells/ml. DMEM containing 0.1% bovine serum albumin (BSA) was used as attractant-free medium (=C_0_). DMEM with 10% FCS was used as chemoattractant. Chemotaxis chambers were homogeneously coated (2 μg/cm^2^) using 16 μg/ml collagen IV (Becton Dickinson, Franklin Lakes, USA) for 30 minutes and allowed to dry prior to use.

For HT-1080 cells, the following experiments were conducted: a negative/negative control with no attractants; a positive/positive control with both reservoirs filled with 10% FCS; and a positive/negative experimental group where only one chamber was filled with attractants. Likewise, the three experiments were created for HUVEC: a negative/negative control with no attractants; a positive/negative group using FCS as attractants; and a group where one reservoir was filled with starved FaDu cells, which produce attractants that mimic the *in vivo *situation more closely. FaDu cells were grown in Dulbecco's modified Eagle's Medium (DMEM; PromoCell, Heidelberg, Germany) without 10% fetal calf serum at 37°C and 5% CO2.

### Cell tracking and image analysis

Video microscopy was performed over a time period of 24 hours when using FCS as chemoattractant or twelve hours when using FaDu cells as attractant source. The time-lapse interval was ten minutes. Cell tracking was performed using the ImageJ software (National Institutes of Health, Bethesda, USA) plugin "Manual Tracking" (Fabrice Cordelières, Institut Curie, Orsay, France). Each experiment was repeated three times, completely independent from each other. On average 40-53 cells were tracked per experiment. In order to further analyse and evaluate chemotactical processes, we developed the ImageJ plugin, which is available for free as a download from the NIH ImageJ homepage. The "Chemotaxis and Migration Tool" provides different types of graphs and statistical tests based on the experimental data. Exported ASCII (mandatory format) tables from "manual tracking" were directly imported into the software tool, and the cell trajectories were all extrapolated to (x, y) = 0 at time 0 h.

For quantification of chemotaxis and migration, several values were generated to evaluate directed cell migration: the centre of mass, the forward migration indices in directions parallel and perpendicular to the direction of the gradient, and the Rayleigh [23] test as well as the cell velocity and directness, where the latter values provide indication for how straight cells move.

### Displacement of centre of mass (COM)

**Figure 1:**
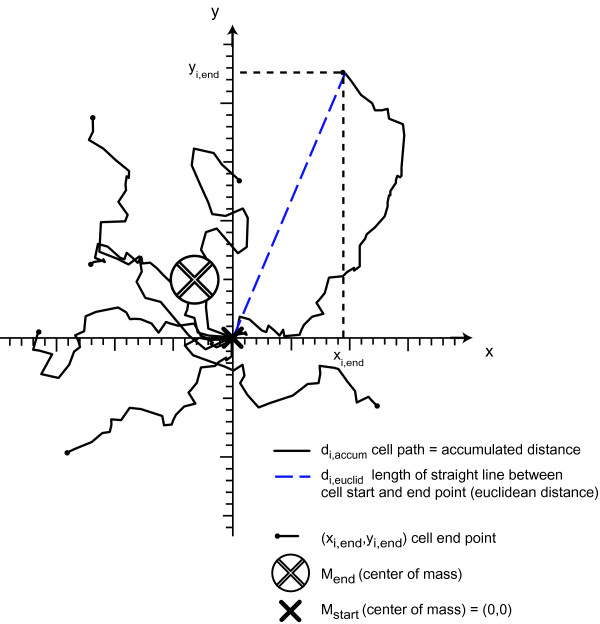
**Trajectory plot defining different parameters for analysing chemotaxis *in vitro *in 2D**. All cell trajectories are transformed by setting each starting point to (x_i,start_, y_i,start_) = (0,0) at time t = 0. 1 ≤ *i *≤ *n *index of the cells.

The centre of mass of all cells was represented by the spatial average of all cell positions. The difference in the centre of mass between its initial value and that at the end of the experiment was termed the displacement of centre of mass (COM, Figure 1), n is the number of analyzed cells.

### Euclidian and Accumulated distances

The Euclidian distance d_i,euclid _for each cell was calculated as the length of the straight line between the cell start and end point. The average Euclidian distance d_euclid _was calculated as the average over all d_i,euclid_.

The accumulated distance d_i,accum _for each cell is the result of the sum of all incremental movements measured in between all m single images. The average accumulated distance d_accum _was calculated from the average over all d_i,accum_, where m is the number of analyzed images (slices) and d_i,j _is the displacement of the cell number i from image j-1 to image j.

### Forward migration indices parallel and perpendicular to the gradient (FMI^║ ^and FMI^┴^)

The FMI^║ ^and FMI^┴ ^[24,25] (Figure 1) represent the efficiency of forward migration of cells parallel and perpendicular to the gradient, respectively. In this publication we chose the coordinate system such that the x-axis was perpendicular to the direction of the gradient and the y-axis was parallel to the gradient.

### Directness (D)

The directness, which represents a measure of the directness of a cell's tendency to travel in a straight line, was calculated by dividing the Euclidian distance by the accumulated distance for each cell. A directness of D ≥ 1 indicates a straight-line migration from start to endpoint. D is the average of all values of directness for all cells.

### Directness of one single cell

### Rayleigh test

The Rayleigh test is a statistical test of the uniformity of a circular distribution of points. The null hypothesis "uniformity with respect to the equal circular distribution of cell endpoints" is rejected with p-values larger than p = 0.05.

### Statistical evaluation

The significance of the experimental data was calculated using a paired student's *t*-test and the Excel software (Microsoft Corp., Redmond, WA).

## Results

### μ-Slide Chemotaxis

The new chemotaxis chamber is designed to be the size of a standard microscopy slide and is manufactured by injection moulding. The bottom is formed as a cover slip-like plastic sheet of 180 μm thickness fitting the standard of No. 1.5 cover slips. The plastic sheet is bonded to the lower side of the slide by a physical melting process, avoiding any use of glue. The material of the chamber was tested for cell culture compatibility. It has extremely low values of birefringence and autofluorescence and a refractive index of n_D _= 1.52, exactly matching that of glass. These characteristics make the μ-Slide Chemotaxis especially suitable for high-resolution microscopy including fluorescence and confocal microscopy.

**Figure 2:**
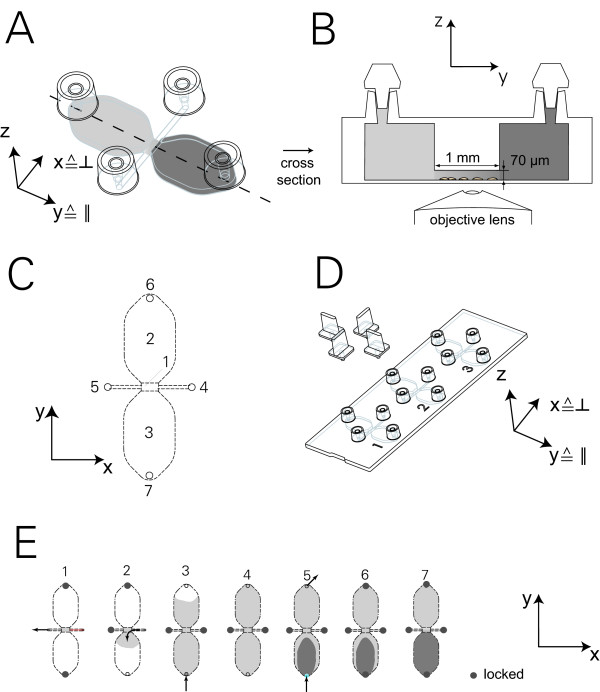
**New chamber format for chemotaxis experiments**. (A) Chamber in 3-D view. The small observation area in the centre connects the two reservoirs along the y-axis. A cross-channel along the x-axis is used to fill cells in the observation area and thereby set up symmetrical initial conditions of cell distribution. The reservoirs are filled either with neutral or chemoattractant solution. (B) Design of the chamber in cross section (not to scale). Adherent cells placed into a small slit become superimposed by a chemoattractant from one side. Optionally, another group of adherent cells can be seeded into one large reservoir to produce chemoattractants. After cell seeding and filling, the chamber is closed by plastic plugs in order to prevent flow due to liquid evaporation. (C) Top view of the chamber with observation area (1), large reservoirs (2, 3), small side channels with filling ports for cell seeding (4, 5) and filling ports for the reservoirs (6, 7). (D) 3D view of the entire slide with three chemotaxis chambers. (E) Picture series showing the handling and filling procedure including cell seeding (1), filling of the entire chamber with chemoattractant-free medium (2 - 4), application of a chemoattractant (5, 6) and the final state for the assay (7).

The design of the chamber consists of two large reservoirs (Figure 2), each with a volume of 40 μl and a height of 0.8 mm. Both reservoirs are connected to a 1 mm wide observation area of 70 μm height and 2 mm length (Figure 2). Two small side channels enable the observation area to be filled with cell suspension. Adherent cells growing in this area become superimposed by a concentration distribution of chemoattractant, which is added into one reservoir via filling ports (Figures 2). One slide with a standard microscopy format consists of three chambers allowing parallel observations when used with an automated stage (Figure 2D).

Setting up gradients with Alexa 488 fluorophore, we found that the fluorescence signal from the dye did not show the expected linear time-stable concentration distribution. Therefore, we added little plugs to hermetically seal the reservoirs in order to avoid any evaporation, which might lead to the expected stability of diffusive concentration distributions.

### Preparation protocol for cell experiments

Preparing the chamber for cell chemotaxis measurements requires that the following aspects be taken into account: (i) the cells should not contact the chemoattractant because this might decrease the cell sensitivity during the experiment; (ii) the trapping of air needs to be avoided because once air bubbles are inside the system they are problematic to remove and disturb the diffusive gradient; (iii) shear forces on the cells during filling of the system need to be minimized in order not to displace the cells. In summary, the following filling protocol proved to be most effective for the μ-Slide Chemotaxis. First, the slide and the medium were degassed by placing them in a standard cell culture incubator. Filling ports 6 and 7 (Figure 2C) were closed with airtight plugs. 6 μl of cell suspension were then placed as a little droplet on top of the filling port 4 of the small cross channel allowing the fluid to be sucked into the observation area through the filling port 5 using a 10 μl pipet. After removing all plugs and covering all four inlet ports with lids, the chamber was placed in a closed 10 cm-Petri dish additionally containing wet paper to prevent evaporation of the small amount of liquid. After two to three hours, cells attached themselves to the incubator chamber. Filling port 6 was then closed using a plug, and reservoir 3 was filled with a chemoattractant-free medium through filling port 4. The plug was then removed from filling port 6 and put into filling port 7. Reservoir 2 was also filled. In order to establish the gradient, filling ports 4 and 5 were closed with plugs, and a droplet of 18 μl of chemoattractant (=C_100_) FCS 10% was put into filling port 6. Exactly 18 μl of liquid were removed from filling port 7, therefore sucking the chemoattractant solution into reservoir 2 (Figure 2E). For positive controls, both reservoirs were filled with a growth medium of concentration C_100_. The chemotaxis setup is indicated as follows: chemotaxis setup where one reservoir is filled with chemoattractant and the other one with neutral medium is referred to as (+/-); the case in which both reservoirs are filled with neutral solution as (-/-); and the case where both reservoirs are filled with chemoattractant as (+/+). All steps were done with caution not to flush out the adhering cells. After closing all inlets, the slide was ready for video microscopy (Figure 2E). For the case using FaDu cells as the attractant source, one reservoir was filled with the cells twelve hours before the start of the experiment.

### Time dependency of gradients within the μ-Slide Chamber

Using confocal imaging, the concentration-distribution of the fluorophore Alexa 488 was measured in the region of the observation area as shown in Figure 2B. Both reservoirs were filled with a C_0 _= 10 μM solution of Alexa 488. Subsequently, 18 μl of a solution with an Alexa 488-concentration of 100 μM was added to only one of the two reservoirs. This procedure resulted in a final concentration of C_100 _= 50.5 μM of fluorophore in the reservoir containing the higher concentration, allowing for measurement of the fluorescence intensity along the observation area with a reasonable signal-to-noise ratio.

**Figure 3:**
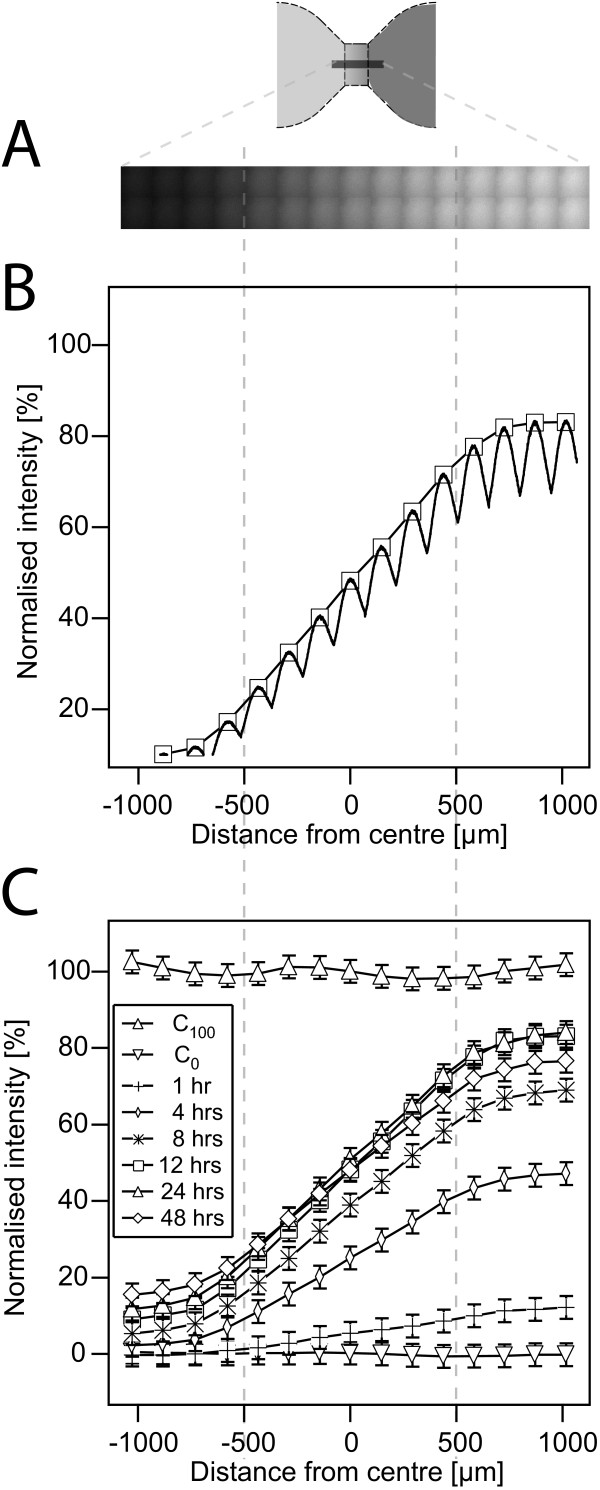
**Fluorescence measurements of concentration profiles by confocal microscopy**. (A) Scan field across the observation area with resulting confocal images stiched together into one large image. (B) Concentration profile in quasi-static conditions after twelve hours. The undulations are due to confocal imaging. The smooth line connects the local maxima of the measured intensity. (C) Time dependence of the concentration profile. Four hours after adding the fluorophore into one of the reservoirs approximately 50% of the overall maximum intensity is reached. The concentration profile changes very little during 48 hours. The size of error bars is twice the standard deviation of the C100 reference measurement. The main error of the repeated experiments is the result of different amounts of "chemoattractant solution" being filled into the reservoir. The amount of liquid filled into the reservoir defines the initial distance between chemoattractant-containing solution and the observation area in which the gradient is to be built.

The confocally measured intensities of the combined scan fields were averaged in the direction perpendicular to the expected gradient (Figure 3A). We found systematic intensity variations due to the pinhole diffraction (Figure 3B). Data smoothing via interpolation only between the local maxima points only revealed a linear increase in intensity from the reservoir containing the lower dye concentration to the other one. Time development showed a shallow gradient at one hour after gradient setup. Steady state was reached after approximately 8-12 hours and lasted at least 48 hours, without significant changes in the concentration distribution (Figure 3C). After two days, the concentration profile slowly flattened due to gradient equalization.

The calculated maximum concentration of 50.5 μM was never observed inside our observation area, probable due to the dilution of fluorophore in the region adjacent to the observation area. By using a reference slide completely filled with dye solution of 50.5 μM, the measured maximum intensity at the edge of the observation areas near the reservoir of higher concentration reached approximately 70% of the intensity of the calibration measurement. In order to attain this condition, it was crucial to prevent any air bubbles from forming inside the reservoirs, filling channel, and filling ports. Air bubbles expand and shrink as temperature changes and induce flow through the only 140 nl containing observation area, disturbing diffusive gradients. The concentration profiles measured over time with Alexa488 are expected to be valid for larger molecules such as most known chemoattractants. With respect to larger molecules, dynamics should by slower due to the typically smaller diffusion constants of larger molecules. Preferably the timescale for other molecules should be calculated using the relation of the diffusion constants. Furthermore, the diffusion constant depends not only on the effective molecule diameter but also on the temperature and dynamic viscosity. For example, use of vascular endothelial growth factor (VEGF) in endothelial cell growth medium instead of Alexa488 in water yields a slower diffusion by a factor of approximately 0.33.

### Direct migration of HT-1080 cells using FCS as chemoattractant

**Figure 4:**
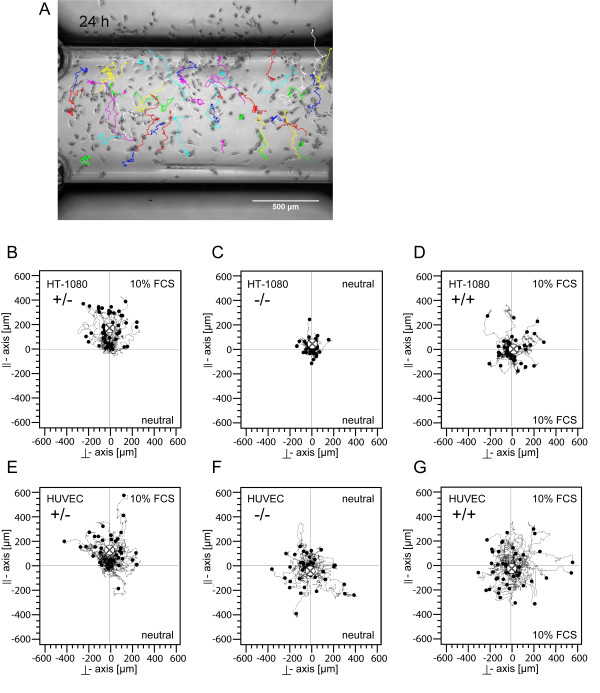
**Microscopic image of the observation area and representative cell trajectory plots**. A: Representative HT-1080 microscopic image of the chamber observation area (2 mm × 1 mm) at time 24 h with an overlay of the tracked cell trajectories. Data from a positive chemotaxis experiment using 10% FCS as attractant (+/-) is shown. Figure 4B shows the corresponding trajectory plot. Figures 4B-D: Representative HT-1080 cell trajectory plots in the time period from 0 to 24 hours. (B) Chemotaxis experiment using 10% FCS as attractant. Cells move in the positive direction of the║-axis, which is by definition the direction of the gradient (+/-). (C) In the negative control, both chambers are filled with FCS-free medium (-/-). Directed cell migration is not visible. (D) In the positive control group, both chambers are filled with FCS 10% (+/+). As in the negative control group, no directed migration can be measured. Cell motility is enhanced. Figures 4E-G: Representative HUVEC trajectory plots in the time period from 0 to 24 hours. (E) Similar to HT-1080, HUVEC also move in the direction of the FCS gradient (+/-). Negative (F, -/-) and positive (G, +/+) controls show no directed migration and a smaller displacement of centre of mass.

In order to distinguish between arbitrary migration effects like enhanced motility and chemotaxis, we made two reference measurements in all cases. For the "negative control" -/-, we observed the migration of the cells in the basic medium without any chemoattractant. For the "positive control", we filled the entire system with chemoattractant solution of 10% FCS. All experiments were repeated at least three times. Data of one experiment are shown. In case of both control measurements, we observed the cells migrating homogeneously in all possible directions (Figures 4C and 4D, respectively). The cell movement in the control measurements can be described as arbitrary and is reminiscent of Brownian motion of small particles in two dimensions. This finding is complemented by the low values of directness of 0.15 and 0.13 for the control measurements (Table 1). In all control measurements, the displacement of the centre of mass was less than 7 μm over 24 hrs (Table 1) in both directions, parallel and perpendicular to the gradient. In comparison, the average accumulated distances of cells were in the range of several hundred micro meters. In the positive control +/+, the average speed was 43.2 μm/hr, and in the negative control -/- it was 18.6 μm/hr. The two control experiments were associated with average values for the Rayleigh test of 0.59 (-/-) and 0.79 (+/+), indicating significant homogeneous distribution of end points of the cell tracks around the origin.

**Table 1:** Averaged parameters of cell migration of all experiments performed.

		Number of experiments	Total number of cells	FMI		COM[μm]/24 hrs		Velocity [μm/hr]	p-Value (Rayleigh test)	Directness
				║	┴	║	┴			
HT-1080	+/-	n = 3	n = 127	0,24	-0,02	139	-7	22,8	3,56703E-08	0,31
	-/-	n = 3	n = 121	0,01	0,01	6	5	18,6	0,59	0,15
	+/+	n = 3	n = 120	0,00	0,01	0	5	43,2	0,79	0,13
HUVEC	+/-	n = 6	n = 194	0,10	0,00	71	-3	31,8	0,01	0,17
	-/-	n = 3	n = 120	-0,03	0,00	-27	1	37,8	0,62	0,16
	+/+	n = 3	n = 125	0,00	0,01	-1	8	48,6	0,50	0,19
	FADU/-	n = 1	n = 50	0,18	-0,06	75	-22	20,9	2,35466E-05	0,44

Each experiment was repeated three times, completely independent from each other. On average 40-53 cells were tracked per experiment. Experiments were highly reproducible.

**Figures 5:**
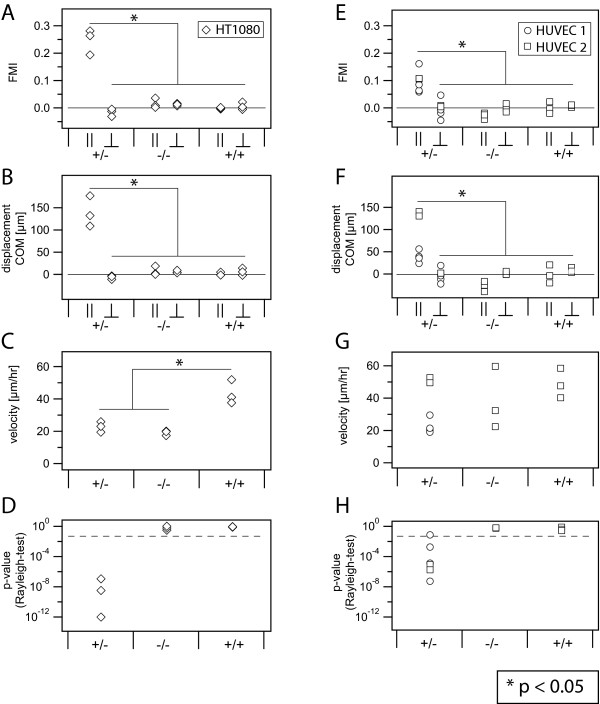
**Analyzed migration parameters**. A-D: Analyzed migration parameters of one set of HT-1080 experiments. Each symbol represents a data point (or its - or ┴-component) of a single measurement. (A) Forward migration indices in parallel (FMI^║^) and perpendicular (FMI^┴^) directions are compared to the values of negative (-/-) and positive controls (+/+). The horizontal line indicates zero. All FMI except FMI^║ ^of the chemotaxis experiment (+/-) are close to 0, indicating arbitrary movements. The FMI^║ ^(+/-) varies between 0.2 and 0.3, representing directed migration. (B) The displacement of the centre of mass (COM) is shown from the same experimental set as A. Like in the case of the FMI, all COM are close to 0, but only the displacement in parallel direction (COM^║^) of the chemotaxis experiment differs strongly from 0. (C) Average cell velocities of HT-1080. In case of the positive control, the velocity is enhanced roughly by a factor of two compared to the chemotaxis experiment (+/-) and the negative control (-/-). (D) Corresponding p-values of the Rayleigh test in logarithmic scale. The dashed line indicates the 0.05 threshold. Only in the (+/-) case is the p-value smaller than 0.05, showing an inhomogeneous cell distribution. Figures 5E-H: Analyzed data of HUVEC cells. Circles (HUVEC 1: self prepared) and squares (HUVEC 2: Lonza) represent parameters of single experiments of two different cell sources. (E) Only the FMI^║ ^of the (+/-) case is clearly larger than 0. No difference between the two different cell sources is visible. (F) COM^║ ^of the (+/-) case of HUVEC 2 is larger than all other COM. (G) Average cell velocities differ dependent on the cell sources. (H) The p-values of the (+/-) data are below 0.05 with one exception. There is no significant difference between the two cell sources.

In Figure 4B, the migration plot of HT-1080 in a gradient of 10% FCS/mm is shown, in which it can be observed that the cells moved in all directions but clearly preferred the direction of the chemoattractant source. The average speed was 22.8 μm/hr, the average accumulated distance travelled was 542 μm within 24 hrs, and the average Euclidian distance was 173 μm in 24 hrs for all cells. The centre of mass shifted COM^║^= 139 μm towards the gradient and COM^┴^= 7.1 μm perpendicular to the gradient. With respect to the control measurements, the directness was enhanced by a factor of two. The forward migration indices parallel and perpendicular to the gradient were FMI^║^= 0.24 and FMI ^┴ ^= - 0.02. The Rayleigh test revealed 3.57· 10^-8^, clearly showing an inhomogeneous distribution of cell end points after 24 hrs of migration with respect to circular distribution. In Figure 5A, the average values of FMI^║ ^and FMI^┴ ^and in Figure 5D corresponding to the results from the Rayleigh test are summarized.

### Using FCS as chemoattractant, HUVEC migrate in the direction of the gradient

We also tested HUVEC from different sources for their chemotactic behavior in gradients of 10% FCS/mm. The trajectory plots are shown in Figures 4E through 4G. In Figures 5E through 5H, data points from HUVEC 1 (self prepared) are represented by circles, whereas data points from HUVEC 2 (Lonza) are shown as squares. All negative -/- and positive +/+ controls were done using HUVEC 2 from Lonza. The chemotaxis experiments +/- and -/- were done with HUVEC 1 and 2 (data from -/- HUVEC 1 not shown). The average cell speed of the positive control was 48.6 μm/hr (+/+) and the negative control was 37.8 μm/hr (-/-). During 24 hrs, the cells migrated on average 907 μm in starving medium (-/-) and 1166 μm in medium containing FCS (+/+). Hence, velocity and motility were increased by the serum. In the case of the chemotaxis experiment +/-, Figure 5G indicates that HUVEC 1 moved with an average velocity of 22.5 μm/hr, much slower than HUVEC 2 with 51.13 μm/hr. The average over both HUVEC preparations of the parallel displacement of mass was COM^║^= 71 μm in 24 hrs for +/-. By a factor of only 2.6, this displacement was larger than the parallel displacement of the centre of mass of the negative control experiment, which was -27 μm. In contrast to inhomogeneous results of COM^║ ^(Figure 5F) and the average velocities (Figure 5G) between HUVEC 1 and HUVEC 2, the values measured for the FMI^║ ^were more consistent. With a value of 0.1, the FMI^║ ^of the chemotaxis experiment was at least three times larger than its FMI^┴ ^and the FMI^║ ^and FMI^┴ ^of the controls for HUVEC (Table1). For the chemotaxis experiment, the values from the Rayleigh tests were smaller than 0.05 with one exception (0.07). In case of the -/- and +/+ controls, the Rayleigh values were always above 0.05. With a value of 0.17, the directness of the chemotaxis experiment was roughly equal to the values of the control measurements (0.16 and 0.19).

**Figure 6:**
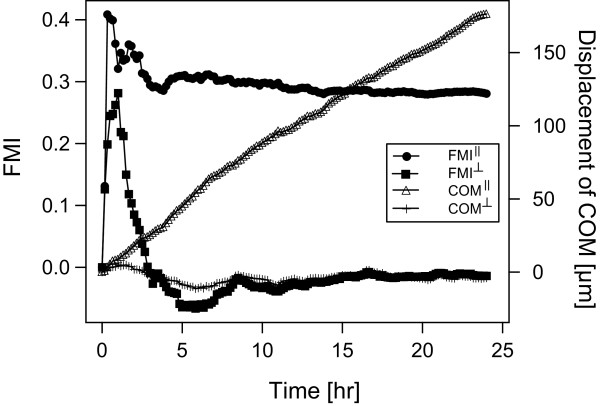
**Time development of ┴ and ║ components of COM and FMI in one representative HT-1080 chemotaxis group (+/-) (data from Figure 4B)**. The FMI^║ ^increases volatile during five hours at the beginning of the experiment and remains nearly constant afterwards. COM^║ ^increases during the whole experimental time with a constant slope. Both, COM^┴ ^and FMI^┴ ^level off around zero indicating no directed migration perpendicular to the direction of the gradient.

In Figure 6, the time dependency of the parallel and perpendicular contributions of the centre of mass and the FMI for HT-1080 cells is shown. The linear increase of the parallel offset of the centre of mass clearly indicates that we observed a systematic effect. Furthermore, the values for the parallel FMI^║ ^and the perpendicular FMI ^┴ ^reached their average values after approximately four hours and remained constant for the rest of the experiment.

### Chemoattractant secreted by FaDu cells cultivated in one reservoir

**Figure 7:**
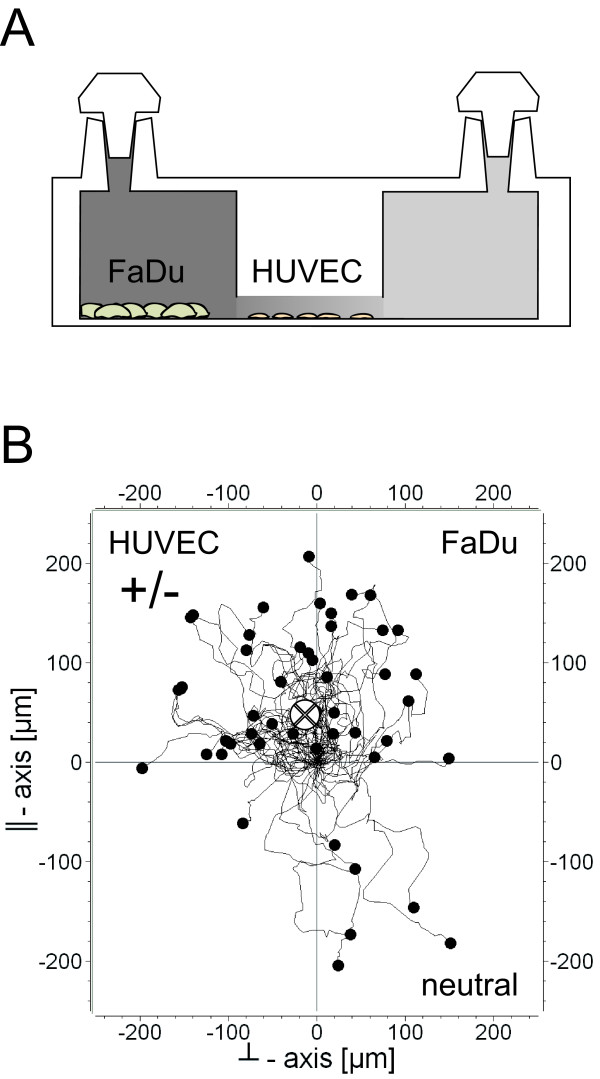
**Optional experimental setup**. (A) A possible setup for testing chemotaxis responses to secreted molecules by another cell type. We chose FaDu cells as a possible source for chemoattractants and tested HUVEC for their chemotactic response. (B) HUVEC show a similar chemotaxis effect in direction toward the FaDu cells, which is comparable to our findings using 10% FCS as chemoattractant.

The third cell system we tested was the chemotactical reaction of HUVEC to molecules secreted by FaDu cells in 96-well plates. In a first attempt, we used medium that was conditioned by FaDu culture over three days. In a recent study, we demonstrated that after three days starved FaDu cells produce 6690 +/- 40.8 pg/ml VEGF and 98 +/- 7 pg/ml bFGF (data not shown see [26]). Building up gradients using the conditioned medium did not lead to observable chemotaxis (data not shown). We excluded possible molecule degradation or dilution effects due to the preparation of the conditioned medium by culturing FaDu cells directly in one of the two reservoirs. Hence, the cells secreted chemoattractant directly in one of the two reservoirs serving as a living chemoattractant source. Interestingly, a strong chemotaxis effect was found in this constellation (Figure 7). The FMI^║^= 0.18 was three times higher than the FMI^┴ ^= 0.06. The Rayleigh test revealed evidence for chemotaxis with a value of 2.35 × 10^-5^. Another important result of this experiment is the finding that it is possible to culture different cell types in the confined geometry of μ-Slide Chemotaxis and still keep the cells in a condition in which they are able to migrate at an undiminished average speed of 20.9 μm/hr.

## Discussion

Chemotaxis is an important field in biology today because it plays a crucial role in neovascularization of solid tumours, metastasis of cancer cells as well as wound healing and many immunological processes. Our chemotaxis approach includes the evaluation of a suitable chamber design for chemotaxis measurements of slow migrating adherent cells like cancer and endothelial cells. The fluorescence measurements of the Alexa 488 concentration clearly show that the chamber design and geometry is well-suited for establishing gradients that remain stable over more than 48 hrs, which is unique for static assays of this kind.

By using plugs, the chamber can be completely sealed and pressure differences relax extremely fast in the system. Inside the small structures, the system is characterized by low Reynolds numbers resulting in a purely diffusive formation of the gradient. In the absence of externally applied pressure, no convection or flow appears as the system is dominated by friction forces.

There is, however, a limitation of the chamber, due to the long time required to establish the gradient, as it takes about 8-12 hours to establish a quasi steady state that is characterized by a linear gradient in the region of the observation area. Fortunately, working with HUVEC and HT-1080 cells, we found that reproducible data can be collected starting immediately after preparing the gradient. The time development shown in Figure 6 indicates that after 3 to 4 hours both FMI components attain their final values, whereas the centre of mass immediately starts to move in direction of the chemoattractant source with rather constant velocity. This finding implies that both kinds of cells are able to sense the gradient during its formation. This result seems less spectacular when keeping in mind that cells in which the absolute cell concentration of the immediate surrounding is low are much more sensitive to spatial concentration changes.

The developed preparation protocol makes observation of chemotaxis of HT-1080 and HUVEC possible, each representing either a commonly used cell line or primary cell type. The ability for co-culturing different cell types allows for testing of the attraction of one cell type by molecules secreted by another cell type. The capability to follow cell migration with video microscopy in combination with the introduced "Chemotaxis and Migration Tool" that is freely available for ImageJ provides access to a complete set of characteristic chemotaxis and migration parameters. We characterize migration of cells in 2D by the parameters average cell velocity and directness and chemotaxis by displacement of centre of mass and forward migration indices in directions both parallel and perpendicular to the direction of the gradient, i.e. FMI^║ ^and FMI^┴^. The "Chemotaxis and Migration Tool" can also be used for various other analysis methods like angle distribution of cell end points, etc.

Our findings show that parameters like the average velocity and the displacement of centre of mass strongly depend on the motility, which is an intrinsic cell property. Only in cases where cells have the same ability to move can parameters be directly compared in a meaningful way. In our example, this condition is not valid for the HUVEC gained from two different sources. We found HUVEC from different sources 1 and 2 migrated with very different average velocities, namely 22.5 μm/hr and 51.1 μm/hr. The first value is nearly in exact agreement with the value of 23 μm/hr given in the literature [27-29]. Generally it is expected that chemokines like VEGF used as chemoattractants also have influence on the motility of cells [30]. From our findings the parameters that appear to be independent with respect to motility include FMI, directness, and the Rayleigh value. Consequently, we conclude that chemotaxis might be best defined through comparison of the parallel FMIs of the Chemotaxis experiment +/- with the perpendicular FMI of +/- and the FMIs of the positive +/+ and negative -/- controls. For basic analysis of chemotaxis, we recommend the use of FMIs and Rayleigh values, as this combination contains sufficient information to characterize chemotaxis. Values like average cell velocity and directness as well as the cell videos allow for detailed observation of the characteristics associated with the movement of cells and cell reaction to reagents. The presence or lack of gradients did not result in any variance in the measured directness of HUVEC cell movement. We therefore conclude that enhancement of directness is not a necessary criterion for evaluation of chemotaxis.

One drawback of video microscopy as a measuring method is the low throughput due to problems that frequently arise when attempting to heavily parallelize the tests. We therefore analyzed the time dependence in order to determine an optimal measurement duration. From Figure 6, we conclude that for HT-1080, twelve hours should be sufficient to analyze the chemotaxis effect. Plotting the time evolution is an effective tool for optimizing the duration of chemotaxis experiments.

There are several viable methods to measure chemotaxis *in vitro*. The Boyden chamber assay is relatively easy to use, requires no expensive equipment and provides quantitative data. Although quantitative assessment of cell migration is possible with a number of methods, the most widely used is the counting of the number of cells that migrate through the filter in several microscopic fields. However, observation of the cells during the migration is not possible, so measurements of cell morphologies and cell speed are not available. The Zigmond-chamber improves on the Boyden design with the decisive advantage of the possibility of microscopy during cell movement accompanied by the disadvantage of complex handling. Consequently, widespread usage is not practical. An easy and economic method is the agarose assay [31], with which the primary benefit is the possibility to observe the chemotaxis with the naked eye without a microscope. Furthermore, it is possible to establish multiple gradients in a single experiment. However, the time-intensive preparations and the instable and poorly defined time gradients are significant disadvantages.

Nevertheless, the Boyden chamber remains the most widely used of all traditional assays. Its two major drawbacks are instability of gradients and poor control over gradient shape in the vicinity of cells. The local gradient may be controlled by the use of point release of chemokines from micropipettes; however, these are only useful for short examinations of fast moving cells before diffusion degrades the gradient and chemokines accumulate in the environment. In Boyden chamber experiments, adherent slow migrating cells are often seeded out to very high densities such that the pores are closed by the cells and chemoattractant is trapped in the pores at high concentration. The cells experience the full concentration of the chemoattractant in the area pointing into the pore but extremely low chemoattrantant concentration in the rest of the cell membrane area. This condition is an accurate simulation of stepwise chemical gradients, which might appear at vessel walls. In contrast, the μ-Slide Chemotaxis is made for simulating shallow gradients, which might appear in interstitial tissue. In this region gradients are formed through chemoattractant sources such as starving tumour cells or inflamed tissue communicating with other cells over distances on the order of a millimeter. The width of the observation area in between the two reservoirs has been chosen to be one mm so that the relative change in concentration over an estimated cell length of 20 μm is always larger than 2%.

Compared to transwell, agarose, or Zigmond chamber assays, microfluidic systems allow for improved control and linearity of chemokine gradients. Furthermore, microfluidic systems are also designed to form various gradient shapes and spatial gradients of different profiles. On the other hand, traditional assays are still preferred because of their simplicity as well as their support for almost every cell type. Additionally, the presence of flow leads in many microfluidic devices result in some significant limitations, and they cannot be used with cells that are sensitive to shear stress. Finally, the microfluidic systems are not easy to use [18].

**Figure 8:**
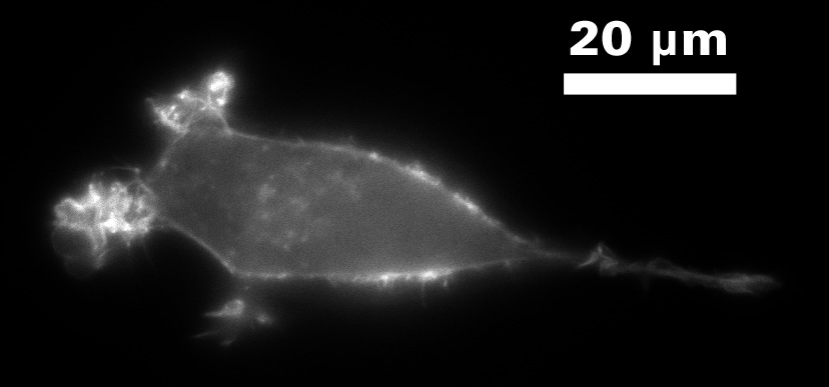
**Single HT-1080 cell, migrating towards a FCS gradient pointing from the right to the left side (higher concentration is on the left side)**. The image was acquired with an epi fluorescence mode using a 60× oil immersion objective (Nikon). Scale bar is 20 μm.

The new chamber described in this publication is easy to handle, allows for the creation of a stable gradient, and permits up to three independent experiments during one examination. Furthermore, life cell microscopy is feasible so that cell movement, cell morphology and cell interaction are observable. Due to the high optical quality (see Figure 8), microscopy measurement with high resolution can also be performed to better evaluate the molecular aspects of cell movement and chemotaxis in chemical gradients.

## Conclusion

The new μ-Slide Chemotaxis described is easy to handle and allows for three independent experiments during one examination. Live cell microscopy is feasible so that cell movement, cell morphology and cell interactions are observable, providing promise that this chamber will facilitate further understanding of chemotaxis.

The μ-Slide Chemotaxis is suitable for identifying new chemoattractants and testing the potential of chemotaxis inhibiting substances. In addition, the influence of different surface coatings can also be investigated. The ability to image cells in gradients with high resolution in combination with fusion proteins like GFP-tubulin or actin labelling peptides like LifeAct [32] opens new possibilities to observe and evaluate migration mechanisms on a molecular level. The future step that we advocate as a potential further benefit involves the transferring of this assay design into a three-dimensional geometry to allow for the observation of cell migration towards attractants in a gel matrix. As recent studies have revealed that migration mechanisms strongly differ in 2D and 3D [33].

## Competing interests

Elias Horn, Valentin Kahl, and Roman Zantl are full-time employees of ibidi. The μ-Slide Chemotaxis is a commercially available product manufactured for performing chemotaxis experiments of slow migrating, adherent mammalian cells. Ibidi has filed a European patent (No EP 05041563) for the μ-Slide chemotaxis and the chemotaxis assay.

Although three of the authors are salaried employees of the ibidi GmbH, all experiments have been objectively performed such that all data are unbiased, accurate and realistic.

## Authors' contributions

PZ designed the study, acquired the data and wrote the manuscript. ANH and CS acquired the data. EH analyzed the data and wrote the manuscript, RZ and VK analysed the data. All authors read and approved the final manuscript.
